# Construction of an artificial biosynthetic pathway for hyperextended archaeal membrane lipids in the bacterium *Escherichia coli*

**DOI:** 10.1093/synbio/ysaa018

**Published:** 2020-09-30

**Authors:** Ryo Yoshida, Hisashi Hemmi

**Affiliations:** Department of Applied Biosciences, Graduate School of Bioagricultural Sciences, Nagoya University, Furo-cho, Chikusa-ku, Nagoya, Aichi 460-8601, Japan

**Keywords:** archaea, isoprenoid, membrane lipid, membrane engineering, prenyltransferase

## Abstract

Archaea produce unique membrane lipids, which possess two fully saturated isoprenoid chains linked to the glycerol moiety via ether bonds. The isoprenoid chain length of archaeal membrane lipids is believed to be important for some archaea to thrive in extreme environments because the hyperthermophilic archaeon *Aeropyrum pernix* and some halophilic archaea synthesize extended C25,C25-archaeal diether-type membrane lipids, which have isoprenoid chains that are longer than those of typical C20,C20-diether lipids. Natural archaeal diether lipids possessing longer C30 or C35 isoprenoid chains, however, have yet to be isolated. In the present study, we attempted to synthesize such hyperextended archaeal membrane lipids. We investigated the substrate preference of the enzyme *sn*-2,3-(digeranylfarnesyl)glycerol-1-phosphate synthase from *A. pernix*, which catalyzes the transfer of the second C25 isoprenoid chain to the glycerol moiety in the biosynthetic pathway of C25,C25-archaeal membrane lipids. The enzyme was shown to accept *sn*-3-hexaprenylglycerol-1-phosphate, which has a C30 isoprenoid chain, as a prenyl acceptor substrate to synthesize *sn*-2-geranylfarnesyl-3-hexaprenylglycerol-1-phosphate, a supposed precursor for hyperextended C25,C30-archaeal membrane lipids. Furthermore, we constructed an artificial biosynthetic pathway by introducing 4 archaeal genes and 1 gene from *Bacillus subtilis* in the cells of *Escherichia coli*, which enabled the *E. coli* strain to produce hyperextended C25,C30-archaeal membrane lipids, which have never been reported so far.

## 1. Introduction

The structure of archaeal membrane lipids is one of the most striking features that distinguishes the organisms of the domain Archaea from those of the domains Bacteria and Eucarya (1, 2). Archaeal membrane lipids with two fully saturated C20 isoprenoid chains that are linked to a glycerol moiety via ether bonds are the major components of membrane in some groups of archaea such as halophilic archaea or exist as minor components in many archaea. These types of archaeal membrane lipids are designated as ‘diether lipids’ because they possess two ether bonds. The production of these lipids is considered an important adaptation mechanism for archaea to thrive in harsh environments such as high temperature and extremely high or low pH, because a cell membrane composed of these lipids maintains a low leakage of ions and small molecules over a wide range of temperatures (3, 4). Interestingly, various archaeal membrane lipids are produced depending on the species of archaea and their growth conditions (5–7). Many thermophilic archaea mainly synthesize tetraether lipids, which are believed to be formed by the dimerization of diether lipids. Some reports have shown that the ratio of tetraether lipids in the cells of thermophilic archaea is increased along with an elevation of the growth temperature, suggesting that these lipids help archaea adapt to more extreme environments (8–10). On the other hand, for some archaea that do not produce tetraether lipids, the length of hydrophobic chains in diether lipids is considered an important feature that allows adaptation to extreme environments. ‘Extended’ C25,C25-archaeal membrane lipids, which have hydrophobic isoprenoid chains that are longer than those of typical C20,C20-diether lipids, are produced in several extremophilic archaea such as the hyperthermophilic archaeon *Aeropyrum pernix* and some halophilic archaea of a few genera, and also in a mesophilic methanogen *Methanomassiliicoccus luminyensis* (11–13). The C25,C25-diether lipids supposedly form a thicker cell membrane that functions as a more impenetrable barrier for ions and small molecules. In fact, the liposomes formed by the C25,C25-archaeal membrane lipids isolated from *A. pernix* are known to maintain low leakage even at 100°C (14). Presumably, archaeal diether lipids with hydrophobic chains that are longer than those of C25,C25-archaeal membrane lipids would form cell membranes that are more stable. Although such ‘hyperextended’ archaeal lipids are yet to be discovered in nature, the physical properties are of great interest.

**Figure 1.**
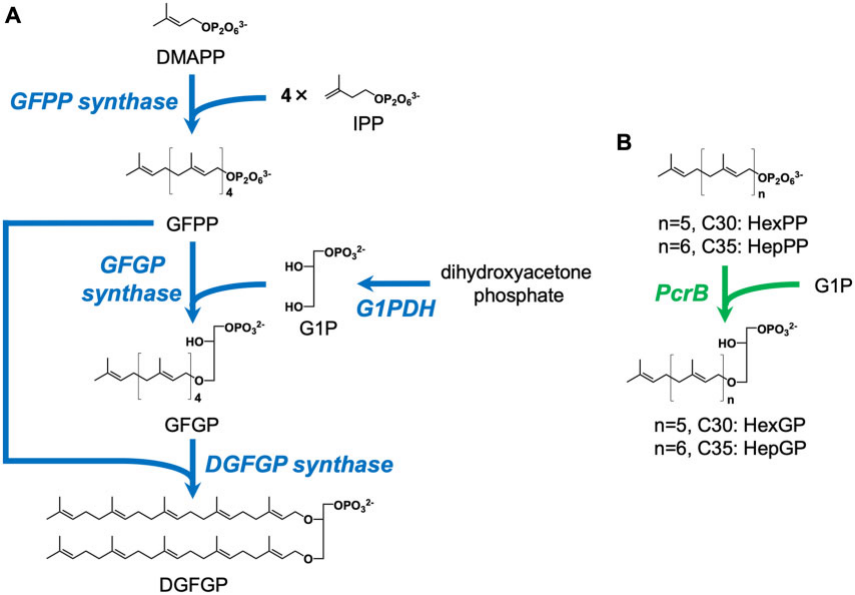
Part of the biosynthetic pathway of extended C25,C25-archaeal membrane lipids in *A. pernix* (**A**) and the reaction catalyzed by PcrB from *B. subtilis* (**B**).

The biosynthetic pathway that yields the hydrocarbon core structure of C25,C25-archaeal membrane lipids has already been identified from *A. pernix* (15) (Figure 1A). First, geranylfarnesyl pyrophosphate (GFPP) is synthesized via GFPP synthase from a molecule of dimethylallyl pyrophosphate (DMAPP) and 4 molecules of isopentenyl pyrophosphate (IPP) (16). Next, *sn*-3-(geranylfarnesyl)glycerol-1-phosphate (GFGP) synthase catalyzes the transfer of a geranylfarnesyl group from GFPP to *sn*-glyceol-1-phosphate (G1P), which is produced by G1P dehydrogenase (G1PDH) from dihydroxyacetone phosphate, to synthesize GFGP. Then, *sn*-2,3-(digeranylfarnesyl)glycerol-1-phosphate (DGFGP) synthase transfers a geranylfarnesyl group from GFPP to the glycerol moiety of GFGP to form DGFGP. Finally, 10 double bonds in the isoprenoid chains of DGFGP are fully reduced by the action of geranylfarnesyl reductase. Therefore, the production of hyperextended diether archaeal lipids, which have isoprenoid chain(s) longer than C25, might be achieved by modification of the biosynthetic pathway for C25,C25-archaeal membrane lipids. Interestingly, *Bacillus subtilis* is known to have a GFGP synthase homolog called PcrB. The enzyme transfers a heptaprenyl (C35) group from heptaprenyl pyrophosphate (HepPP) to G1P to form *sn*-3-heptaprenylglycerol-1-phosphate (HepGP), the physiological function of which remains unclear (17–19) (Figure 1B). We speculated that if DGFGP synthase could accept prenyl donor and acceptor substrates with longer chains, such as HepPP and HepGP, respectively, utilization of PcrB and suitable prenyl pyrophosphate synthases could enable the synthesis of hyperextended archaeal membrane lipids. A membrane intrinsic prenyltransferase, UbiA, which is involved in ubiquinone biosynthesis, catalyzes the transfer of a C30-50 prenyl chain from prenyl pyrophosphate to *p*-hydroxybenzoate. Structural analysis of the *A. pernix* UbiA homolog (20) has demonstrated the existence of a ‘lateral portal’ that faces the hydrophobic region of a cell membrane and allows the enzyme to accept a long prenyl pyrophosphate as a prenyl donor substrate, while the UbiA homolog from *Archaeoglobus fulgidus* (21) has a large internal cavity that could likely accommodate prenyl pyrophosphate. Because *A. pernix* DGFGP synthase belongs to the UbiA superfamily and has a sequence that approximates that of the *A. pernix* UbiA homolog (30% identity), we expected this enzyme to accept longer prenyl donor substrates such as HepPP.

In the present study, we attempted to construct artificial biosynthetic pathways for hyperextended archaeal diether lipids with hydrophobic chains that would be longer than those of the C25,C25-archaeal membrane lipids. Contrary to our expectations based on the characteristics of the UbiA-superfamily prenyltransferases, however, *in vitro* study of the substrate preferences of *A. pernix* DGFGP synthase revealed that this enzyme does not accept longer (>C25) prenyl donor substrates. The enzyme, however, could accept a longer prenyl acceptor substrate, *sn*-3-hexaprenylglycerol-1-phosphate (HexGP), which allowed the synthesis of a supposed precursor for hyperextended C25,C30-archaeal membrane lipids. We constructed a plasmid containing 4 archaeal genes and the *B. subtilis* PcrB gene, which was sufficient to produce the precursor in *E. coli* cells. An *E. coli* strain harboring the plasmid produced unsaturated C25,C30-archaeal membrane lipids with diacylglycerol- and phosphatidylglycerol-like structures.

## 2. Materials and methods

### 2.1 Materials

Precoated reversed-phase thin-layer chromatography (TLC) plates, silica gel RP-18 F_254S_, were purchased from Merck Millipore, Germany. [1-^14^C]IPP (55 Ci/mol) was purchased from American Radiolabeled Chemicals, Inc., USA. Non-labeled IPP and DMAPP were donated by Dr. Chikara Ohto, Toyota Motor Co., Japan.

### 2.2 General procedures

Restriction enzyme digestions, transformations, and other standard molecular biological techniques were carried out as described by Sambrook *et al.* (22).

### 2.3 Cultivation of microorganisms and extraction of their genomes

*B. subtilis* and *Saccharolobus solfataricus* (former *Sulfolobus solfataricus*) were provided by the RIKEN BRC through the Natural Bio-Resource Project of the MEXT, Japan. *B. subtilis* was cultured in a LB medium at 37°C. *S. solfataricus* was cultured in an ATCC1304 *S. solfataricus* medium at 70°C. The genomic DNAs of *B. subtilis* and *S. solfataricus* were extracted from cells using a DNA extraction kit, Geno Plus™ Mini (VIOGENE, USA).

### 2.4 Recombinant expression and purification of *B. subtilis* PcrB and archaeal enzymes

All plasmids used in this study are listed in Table 1. PCR reactions were performed using KOD plus NEO DNA polymerase (TOYOBO, Japan) and the primers shown in Table 2. The *BSU06600* gene encoding *B. subtilis* PcrB was amplified using the genomic DNA of *B. subtilis* as a template. The amplified gene was digested by restriction enzymes *Nde*I and *Bam*HIl, and was then ligated into pET15b digested by the same restriction enzymes to construct pET15b-BsPcrB. *E. coli* BL21(DE3) transformed by the plasmid was cultivated at 37°C in 1 l LB medium supplemented with 100 mg/l ampicillin. When the culture reached an optical density of 0.5, 1.0 mM IPTG was then added for induction. After an additional 24 h of incubation, the cells were harvested and disrupted by sonication in a HisTrap binding buffer that contained 20 mM potassium phosphate, pH 7.4, 0.5 M NaCl and 10 mM imidazole. The homogenate was centrifuged at 4000 g for 30 min, and the supernatant was recovered as a crude extract. The supernatant fraction was loaded into a HisTrap crude FF column (GE Healthcare, USA), which had been equilibrated with HisTrap binding buffer. The column was washed with HisTrap wash buffer containing 20 mM potassium phosphate, pH 7.4, 0.5 M NaCl and 60 mM imidazole. Then, the recombinant proteins were eluted with a HisTrap elution buffer containing 20 mM potassium phosphate, pH 7.4, 0.5 M NaCl and 500 mM imidazole, to be used for the synthesis of radiolabeled substrates. The level of purification was confirmed by SDS-PAGE.

**Table 1.** Plasmids used in the study.

**Plasmid** ^a^	Characteristics	References
pET-15b	Amp^R^, pBR322_origin, P_T7_, His-tag	Novagen
pET-HisGGPS	Amp^R^, pBR322_origin, P_T7_, His-tag, *Saci0092*	(23)
pET-15b-gfps	Amp^R^, pBR322_origin, P_T7_, His-tag, *APE1764*	(24)
pET-PTH	Amp^R^, pBR322_origin, P_T7_, *SSO2345*	(25)
pET3a-AF1551	Amp^R^, pBR322_origin, P_T7_, *AF1551*	(26)
pET48b-APE0621	Kan^R^, pBR322_origin, P_T7_, Trx-tag, His-tag, *APE0621*	(15)
pET15b-APE0159	Amp^R^, pBR322_origin, P_T7_, His-tag, *APE0159*	(15)
pET15-BsPcrB	Amp^R^, pBR322_origin, P_T7_, His-tag, *BSU06600*	This study
pBAD18	Amp^R^, pBR322_origin, P_ARA_	(27)
pBAD-MA3686	Amp^R^, pBR322_origin, P_ARA_, *MA3686*	(28)
pBAD-C25ALB4	Amp^R^, pBR322_origin, P_ARA_, *APE0159*, *APE1764*, *APE0621*, *MA3686*	(15)
pBAD-C30ALB2	Amp^R^, pBR322_origin, P_ARA_, *BSU06600*, *MA3686*	This study
pBAD-C30ALB3	Amp^R^, pBR322_origin, P_ARA_, *SSO2345*, *BSU06600*, *MA3686*	This study
pBAD-C30ALB5	Amp^R^, pBR322_origin, P_ARA_, *APE0159*, *APE1764*, *SSO2345*, *BSU06600*, *MA3686*	This study

^a^ The fully annotated sequences of the plasmids used in this study are provided as Supplementary Data.

**Table 2.** Primers used in the study

Gene	Primer sequence	Constructed plasmid
***BSU06600***	forward: 5′-CAGCTCATATGTACGATGTAACGGAGTGG-3′ reverse: 5′-ACTGTGGATCCTTACTCGCCTTTCACAGCC-3′	pET15b-BsPcrB
***BSU06600***	forward: 5′-TTTTTTTGGGCTAGCGAATTCAAGAAGATTATTATGTACGATGTAACGGAGTGG-3′ reverse: 5′-TTTTTATTTGAGCTCGAATTATTACTCGCCTTTCACAGCC-3′	pBAD-C30ALB2
***SSO2345***	forward: 5'-TTTTTTTGGGCTAGCGAATTCAGGAGAAATATAATGAGTATTATAGAGTTCTGGTTAGAGGC-3′ reverse: 5′-CATAATAATCTTCTTGAATTATTAAATCTTATCTATGTTAGCCTCCTTTAG-3′	pBAD-C30ALB3
***APE0159*** and ***APE1764***	forward: 5'-TTTTTTTGGGCTAGCGAATTCAAGAAGATATAAATGAAGGCTGCTATCGAGATAACTAGG-3′ reverse: 5′-CATTATATTTCTCCTGAATTATTACTTCTCCCTCTCCACAATATAGTCTAGAAG-3′	pBAD-C30ALB5

For expression and purification of *S. solfataricus* hexaprenyl pyrophosphate (HexPP) synthase and *A. fulgidus* HepPP synthase, the *E. coli* BL21(DE3) strain was transformed using pET-PTH, which includes the gene that encodes *S. solfataricus* HexPP synthase (25), and pET3a-AF1551 that encodes *A. fulgidus* HepPP synthase (26), respectively, via cultivation at 37°C in 1 l LB medium supplemented with 100 mg/l ampicillin. When the culture reached an optical density of 0.5, then 1.0 mM IPTG was added for induction. After an additional 24 h of incubation, the cells were harvested and disrupted by sonication in 50 mM 3-(*N*-morpholino)propanesulfonic acid (MOPS)-NaOH buffer, pH 7.0. The homogenate was centrifuged at 4000 *g* for 30 min, and each supernatant was recovered as a crude extract. The crude extracts were heated at 55°C for 30 min, and the denatured proteins were removed by centrifugation at 4000 *g* for 30 min. The supernatant fractions were used for the synthesis of radiolabeled substrates.

Purified *Sulfolobus acidocaldarius* geranylgeranyl pyrophosphate (GGPP) synthase, *A. pernix* GFPP synthase, GFGP synthase, and DGFGP synthase were prepared as described elsewhere (15, 23,24).

### 2.5 Synthesis of radiolabeled substrates for radio-TLC assay

[^14^C]GFPP was prepared from DMAPP and [^14^C]IPP (American Radiolabeled Chemicals, Inc., USA) using *A. pernix* GFPP synthase as described in our previous report (24). For the synthesis of [^14^C]HexPP and [^14^C]HepPP, we first prepared [^14^C]GGPP using *S. acidocaldarius* GGPP synthase from DMAPP and [^14^C]IPP as described in our previous study (23). The resultant 1-butanol solution containing ∼56 pmol [^14^C]GGPP (corresponding to 20 000 dpm) then was dried under a stream of N_2_ gas. To dissolve the residue, we added 200 µl of 0.1 M MOPS-NaOH buffer, pH 7.0, containing 0.2 mmol MgCl_2_, 0.1% Triton-X, 0.2 nmol IPP and a suitable amount of either *S. solfataricus* HexPP synthase or *A. fulgidus* HepPP synthase. The mixtures were incubated at 55°C for 60 min. The products were extracted with 1-butanol saturated with water and used as substrates for the reaction of *A. pernix* DGFGP synthase. A radiolabeled prenyl acceptor substrate for *A. pernix* DGFGP synthase, [^14^C]GFGP, was prepared using *A. pernix* GFGP synthase as described in our previous report (15). For the syntheses of [^14^C]*sn*-3-hexaprenylglycerol-1-phosphate (HexGP) and [^14^C]*sn*-3-heptaprenylglycerol-1-phosphate (HepGP), a 1-butanol solution containing ∼28 pmol of [^14^C]HexPP or [^14^C]HepPP (both corresponding to 10 000 dpm) was dried under a stream of N_2_ gas. Then, 200 µl of 0.1 M MOPS-NaOH buffer, pH 7.0, containing 0.2 mmol MgCl_2_, 10 nmol α-glycerophosphate (racemic mixture) and a suitable amount of the purified *B. subtilis* PcrB was added to dissolve the residue. These mixtures were incubated at 37°C for 150 min, and the products were extracted with 1-butanol saturated with water and used as substrates for the reaction of *A. pernix* DGFGP synthase. The concentrations of the radiolabeled substrates were determined by measuring radioactivity with an LSC-5100 liquid scintillation counter (ALOKA, Japan). To confirm the formation of the expected radiolabeled substrates, each substrate was treated with potato acid phosphatase (SigmaAldrich, USA) according to a method established by Fujii *et al.* (29), and their hydrolysates were extracted with *n*-pentane to be analyzed by reversed-phase TLC using a precoated plate, silica gel RP-18 F_254S_ (Merck Millipore, Germany) developed with acetone/H_2_O (19:1). The distribution of radioactivity on the TLC plate was visualized using a Typhoon FLA9000 multifunctional scanner (GE Healthcare, USA).

### 2.6 Radio-TLC assay for DGFGP synthase

To know if *A. pernix* DGFGP synthase accepts C30 or C35 prenyl donor and acceptor substrates, a prenyl donor substrate, ∼10 pmol [^14^C]GFPP or ∼14 pmol [^14^C]HexPP or ∼14 pmol [^14^C]HepPP and a prenyl acceptor substrate, ∼10 pmol [^14^C]GFGP or ∼14 pmol [^14^C]HexGP or ∼14 pmol [^14^C]HepGP, each corresponding to 5000 dpm, were mixed and dried under a stream of N_2_ gas. Then 200 µL of 0.1 M MOPS-NaOH buffer, pH 7.0, containing 0.2 mmol MgCl_2_, 0.3 nmol 3-[(3-cholamidopropyl)dimethylammonio]propanesulfonate, and a suitable amount of purified *A. pernix* DGFGP synthase was added to dissolve the residue. These mixtures were incubated at 60°C for 30 min, and the products were extracted with 1-butanol saturated with water. The products were analyzed by reversed-phase TLC following phosphatase treatment as described above.

### 2.7 Construction of a biosynthetic pathway of hyperextended archaeal membrane lipids in *E. coli*

For the construction of a plasmid containing five genes sufficient for the biosynthesis of C25,C30-archaeal membrane lipids in *E. coli*, the *BSU6600* gene encoding *B. subtilis* PcrB was amplified using pET15b-BsPcrB as a template, KOD plus NEO DNA polymerase and the primers shown in Table 2. The amplified gene and *EcoR*I-digested pBAD-MA3686, which contains the gene of G1PDH from *Methanosarcina acetivorans* (28), were introduced into the *E. coli* ME9783 strain (National BioResource Project, Japan), which enables *in vivo E. coli* cloning based on homologous recombination (30). The plasmid in which the *BSU6600* gene was inserted was extracted from an *E. coli* clone and was designated pBAD-C30ALB2. In a similar manner, a *SSO2345* gene encoding *S. solfataricus* HexPP synthase was amplified using the genomic DNA of *S. solfataricus* as a template and was inserted into *EcoR*I-digested pBAD-C30ALB2 to construct pBAD-C30ALB3. Next, a DNA fragment containing *APE0159* and *APE1764* genes encoding *A. pernix* DGFGP synthase and GFPP synthase, respectively, was amplified from pBAD-C25ALB4, which had been constructed in our previous report for the biosynthesis of C25,C25-archeal membrane lipids in *E. coli* (15). The DNA fragment was inserted into *EcoR*I-digested pBAD-C30ALB3 to construct pBAD-C30ALB5.

An *E. coli* TOP10 strain transformed by pBAD-C30ALB5 or an empty plasmid, pBAD-18 (27), was cultivated at 37°C for 24 h in 1 l of LB medium supplemented with 100 mg/l ampicillin and 0.02% L-arabinose. After cultivation, cells were harvested and then dissolved with 10 ml of 1-butanol/75 mM ammonium water/ethanol (4:5:11) per 1 g of wet cells. The mixture was heated to 70°C and shaken vigorously for 1 min. After cooling to room temperature, the mixture was centrifuged at 1000 *g* for 15 min. The supernatant was recovered and dried under a N_2_ stream. The residue was dissolved with 5.2 ml of 1-butanol/methanol/0.5 M acetate buffer, pH 4.6, (3:10:5) per 1 g of wet cells. Lipids in the mixture were extracted twice with 3 ml *n*-pentane and dried under a N_2_ stream. The residue was dissolved with 0.5 ml of methanol/2-propanol (1:1) per 1 g of wet cells.

The lipid extracts were analyzed by LC-ESI-MS using an Esquire 3000 ion trap system (Bruker Daltonics, USA) equipped with an Agilent 1100 Series HPLC system (Agilent Technologies, USA). The parameters for MS were the same as those described in our previous report (28). A 10 µl of each lipid extract was injected into a COSMOSIL 5C_18_-AR-II packed column (2.0 mm × 150 mm, Nacalai, Japan) and eluted at a flow rate of 0.2 ml/min with eluent A: methanol/10 mg/l sodium acetate (9:1) and eluent B: 2-propanol. The procedure used was 100% of eluent A for 0-20 min, a linear gradient with 0–80% of eluent B for 20–50 min and 80% of eluent B for 50–70 min.

### 2.8 Availability of materials and resources

Any unique materials and resources presented in the manuscript may be available from the authors upon reasonable request and through a materials transfer agreement.

## 3. Results

### 3.1 Synthesis of radiolabeled substrates for DGFGPS assay

**Figure 2.**
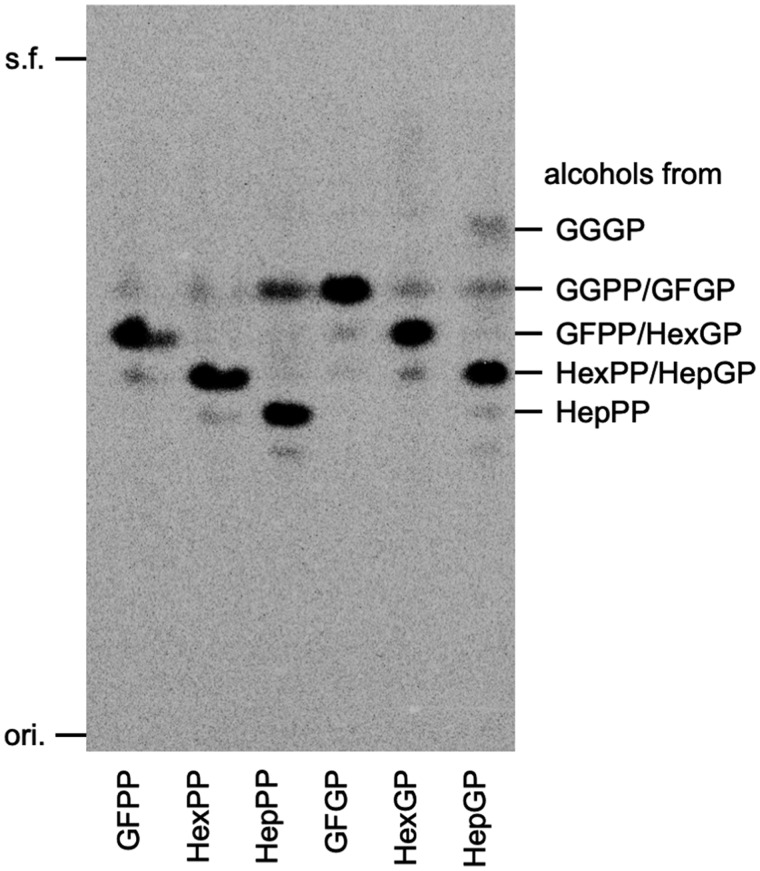
The radio-TLC analysis of enzymatically synthesized prenyl donor and acceptor substrates. Synthesized prenyl donors and acceptors for the DGFGP synthase reaction, which were extracted from reaction mixtures with 1-butanol, were hydrolyzed with acid phosphatase and then analyzed by reversed-phase TLC after pentane extraction. Radiolabeled GFPP was synthesized from non-labeled DMAPP and [^14^C]IPP. Radiolabeled HexPP and HepPP were synthesized from [^14^C]GGPP and non-labeled IPP. Radiolabeled GFGP, HexGP, and HepGP were synthesized from G1P and corresponding prenyl pyrophosphate, i.e., [^14^C]GFPP, [^14^C]HexPP, or [^14^C]HepPP, respectively. ori., origin; s.f., solvent front.

For the *in vitro* assay of DGFGP synthase, we prepared [^14^C]GFPP, [^14^C]HexPP and [^14^C]HepPP as candidates for prenyl donor substrates, and [^14^C]GFGP, [^14^C]HexGP and [^14^C]HepGP as possible prenyl acceptor substrates. GFPP was synthesized using *A. pernix* GFPP synthase from non-labeled DMAPP and [^14^C]IPP. HexPP and HepPP were synthesized using *S. solfataricus* HexPP synthase, and *A. fulgidus* HepPP synthase, respectively, from [^14^C]GGPP and non-labeled IPP. The formation of the substrates was confirmed by radio-TLC analysis following phosphatase treatment. As shown in Figure 2, the radioactive spots that corresponded to C25, C30 or C35 prenyl alcohol emerged, confirming that radiolabeled GFPP, HexPP and HepPP were synthesized as major products. In the analysis of [^14^C]HepPP, a faint spot corresponding to geranylgeraniol, which arose from unreacted [^14^C]GGPP, was observed. The amount of unreacted GGPP estimated from the density of the spot was, however, much lower than that of HepPP, suggesting that most of the GGPP was converted to HepPP. To synthesize the prenyl acceptor substrates, *A. pernix* GFGP synthase and its homolog prenyltransferase PcrB from *B. subtilis* were expressed in *E. coli* and purified. [^14^C]GFGP was successfully synthesized using GFGP synthase. To synthesize [^14^C]HexGP and [^14^C]HepGP, we reacted [^14^C]HexPP and [^14^C]HepPP, respectively, with PcrB in the presence of an excess amount of G1P. Hydrophobic products and unreacted substrates extracted from the reaction mixture were analyzed by reversed-phase TLC following phosphatase treatment. As shown in Figure 2, new spots with values for *R*_f_ of 0.59 and 0.53 were considered to emerge from alcohols from HexGP and HepGP, respectively, because of the disappearance of the spots of the alcoholic forms of HexPP and HepPP that had values for *R*_f_ of 0.53 and 0.47, respectively, and because a similar level of increase in the value of *R*_f_ was also observed between the alcohols from GFPP (*R*_f_ 0.59) and GFGP (*R*_f_ 0.65). These results suggested that HexPP and HepPP were almost completely consumed to synthesize HexGP and HepGP, respectively.

### 3.2 *In vitro* assay of *A. pernix* DGFGP synthase

**Figure 3.**
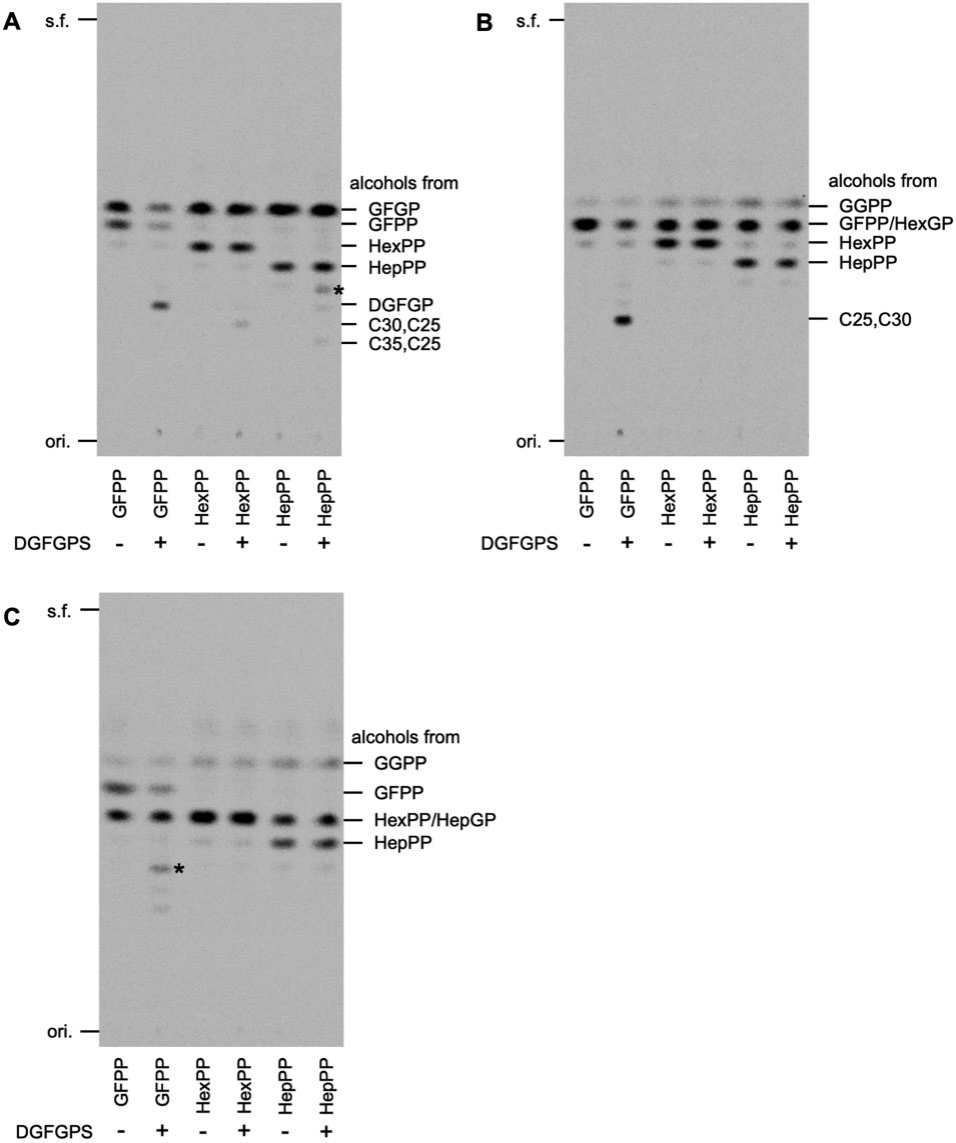
The radio-TLC analysis of the products of DGFGP synthase from various prenyl donor and acceptor substrates. The butanol-extracted products were hydrolyzed with acid phosphatase and analyzed by reversed-phase TLC after pentane extraction. Asterisks indicate the products from unexpected reactions as explained in the main text. s.f., solvent front; ori., origin; DGFGPS, A. *pernix* DGFGP synthase. (**A**) Analysis of the products from [^14^C]GFGP and various prenyl donor substrates, i.e., [^14^C]GFPP, [^14^C]HexPP, and [^14^C]HepPP. C30,C25 and C35,C25 indicate *sn*-2-hexaprenyl-3-(geranylfarnesyl)glycerol-1-phosphate and *sn*-2-heptaprenyl-3-(geranylfarnesyl)glycerol-1-phosphate, respectively. (**B**) Analysis of the products from [^14^C]HexGP and each prenyl donor substrate. C25,C30 indicates *sn*-2-geranylfarnesyl-3-hexaprenylglycerol-1-phosphate. (**C**) Analysis of products from [^14^C]HepGP and each prenyl donor substrate.

To test the capability of DGFGP synthase to accept substrates longer than the original C25 substrates GFPP and GFGP, recombinant DGFGP synthase was prepared by purification via heat treatment and affinity column chromatography. First, DGFGP synthase was reacted with the original prenyl acceptor substrate [^14^C]GFGP and various prenyl donors: [^14^C]GFPP, [^14^C]HexPP and [^14^C]HepPP. Radio-TLC analysis of the reaction products extracted with 1-butanol was performed following phosphatase treatment. As shown in Figure 3A, the spot of DGFGP-derived alcohol with an *R*_f_ of 0.32 emerged when GFPP and GFGP were used, confirming the occurrence of the original DGFGP synthase reaction. As HexPP was used instead of GFPP, a new faint spot with an *R*_f_ of 0.28 emerged. Because the difference in *R*_f_ between this spot and that corresponding to alcohol from HexPP was comparable to that observed in the analysis of DGFGP and GFPP, this result suggested the formation of *sn*-2-hexaprenyl-3-(geranylfarnesyl)glycerol-1-phosphate, a precursor for C30,C25-archaeal membrane lipids, by prenyltransfer reaction from HexPP to GFGP. However, the amount of the reaction product estimated from the density of the spot was much lower than that of DGFGP. When HepPP was used as the prenyl donor substrate, two new spots were detected. The faint spot with an *R*_f_ of 0.38 that is indicated by the asterisk in Figure 3A, likely corresponded to the alcoholic form of *sn*-2-geranylgeranyl-3-(geranylfarnesyl)glycerol-1-phosphate synthesized from GGPP, which had been mixed in the solution of HepPP as shown in Figure 2, and GFGP. The other very faint spot with an *R*_f_ of 0.25 was considered to arise from the alcoholic form of *sn*-2-heptaprenyl-3-(geranylfarnesyl)glycerol-1-phosphate, suggesting that the heptaprenyl group was transformed to GFGP to form the precursor of C35,C25-archaeal membrane lipids, while the amount of the product was almost negligible compared with that of the original reaction.

Next, DGFGP synthase was reacted with [^14^C]HexGP, as the prenyl acceptor substrate, and each of the donor substrates. When HexGP and GFPP were used for the reaction, a new dense spot with an *R*_f_ of 0.28 arose, suggesting the formation of *sn*-2-geranylfarnesyl-3-hexaprenylglycerol-1-phosphate, a precursor of C25,C30-archaeal membrane lipids (Figure 3B). The density of the spot demonstrated that the amount of the reaction product was comparable to that of DGFGP from the original reaction with GFPP and GFGP. When either HexPP or HepPP was used instead of GFPP, no new spot was detected. These results demonstrate that DGFGP synthase is capable of accepting HexGP as the effective prenyl acceptor only when the donor substrate is GFPP.

Finally, DGFGP synthase was reacted with [^14^C]HepGP and each of the prenyl donor substrates. When GFPP was used for the reaction, a faint spot with an *R*_f_ of 0.40 was observed (Figure 3C, shown by an asterisk). The spot did not correspond to the alcoholic form of the expected product from the reaction between GFPP and HepGP, but possibly did correspond to that of the *sn*-2-geranylfarnesyl-3-(geranylgeranyl)glycerol-1-phosphate that was synthesized from GFPP and GGGP, which had been mixed to a small extent into the solution of HepGP (Figure 2). With HexPP and HepPP, no new spot was observed. These results suggested that DGFGP synthase is incapable of accepting HepGP as the prenyl acceptor.

Contrary to our expectations, the results from *in vitro* enzyme assay showed that DGFGP synthase has a relatively specific substrate preference, particularly for its donor substrate. The fact that the enzyme effectively catalyzes the reaction between GFPP and HexGP, however, suggests the possibility that an artificial synthetic pathway of hyperextended C25,C30-archaeal membrane lipids could yet be constructed.

### 3.3 Construction of an artificial biosynthetic pathway of hyperextended archaeal membrane lipids in *E. coli*

**Figure 4.**
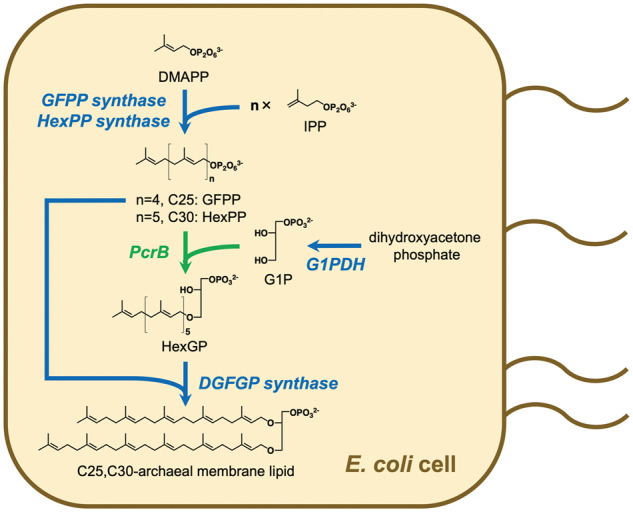
The artificial biosynthetic pathway of hyperextended C25,C30-archaeal membrane lipids constructed in this study.

**Figure 5.**
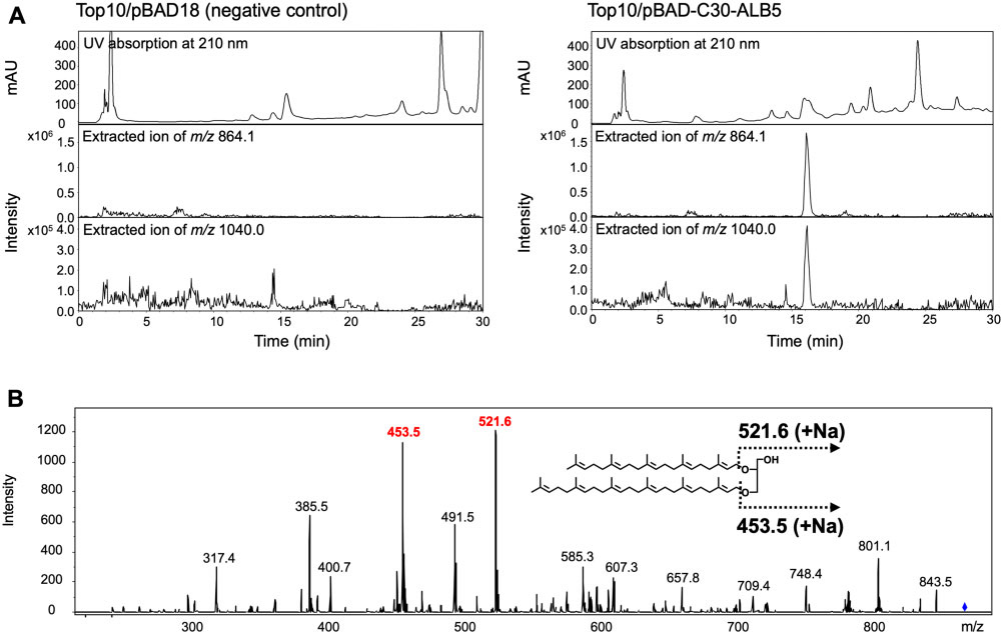
LC-ESI-MS analysis of lipids extracted from *E. coli* strains. (**A**) Analysis of the lipid samples extracted from *E. coli* harboring the empty vector pBAD18 (left panels), as the negative control, and pBAD-C30ALB5 (right panels). UV chromatogram at 210 nm is shown in the top panel. The extracted ion chromatogram of *m/z* 864.1 corresponding to [C25,C30-OH + Na]^+^ is shown in the middle panel. The extracted ion chromatogram of *m/z* 1040.0 corresponding to [C25,C30-PG + 2Na]^+^ is shown in the bottom panel. (**B**) MS/MS analysis of the ion with *m/z* of 864.1 in the lipid sample extracted from *E. coli* harboring pBAD-C30ALB5. Inset: Predicted fragmentation patterns corresponding to the major fragment ions are depicted.

To construct the artificial biosynthetic route for C25,C30-archaeal membrane lipids in *E. coli* cells, we introduced the genes of *A. pernix* DGFGP synthase and GFPP synthase, *S. solfataricus* HexPP synthase, *B. subtilis* PcrB and *M. acetivorans* G1PDH into the same plasmid to construct pBAD-C30ALB5 (Figure 4). Lipids extracted from the *E. coli* strain harboring the plasmid were analyzed via LC-ESI-MS. As shown in Figure 5A, a positive ion peak with an *m/z* of 864.1, which was not observed in the analysis of the negative control sample, was eluted at ∼16 min of retention time. This suggests that a diacylglycerol-like C25,C30-archeal membrane lipid (C25,C30-OH), i.e., *sn*-2-geranylfarnesyl-3-hexaprenylglycerol, actually was produced because the *m/z* value corresponded to [C25,C30-OH + Na]^+^. To confirm the production of C25,C30-OH, we performed LC-ESI-MS/MS analysis for this ion. Detected major fragment ions with *m/z* values of 521.6 and 453.5 could be reasonably explained via the conceivable fragmentation of C25,C30-OH, as shown in Figure 5B. In addition, a positive ion peak with an *m/z* of 1040.0 was eluted with the same retention time, which suggested that actually a phosphatidylglycerol-like C25,C30-archaeal membrane lipid (C25,C30-PG), i.e., *sn*-2-geranylfarnesyl-3-hexaprenylglycerol-1-phosphoglycerol was produced because this *m/z* value corresponded to [C25,C30-PG + 2Na]^+^. The identical elution time of the two ion peaks suggests that C25,C30-OH might arise from the decomposition of C25,C30-PG through ionization.

## 4. Discussion

In the present study, an *in vitro* assay of *A. pernix* DGFGP synthase was performed in an effort to construct artificial biosynthetic pathways for hyperextended archaeal membrane lipids. Although *A. pernix* DGFGP synthase could not accept longer prenyl donors such as HexPP and HepPP as proper substrates, the enzyme efficiently accepted the C30 prenyl acceptor substrate HexGP when GFPP was the donor substrate. This enzyme belongs to the UbiA superfamily, and therefore is considered to have a structure similar to UbiA, which is a bacterial prenyltransferase involved in ubiquinone biosynthesis (31). Structural analysis has suggested that the *A. pernix* UbiA homolog, which is presumably responsible for the biosynthesis of *A. pernix*-specific demethylmenaquinones or methionaquinones with a fully/partially saturated C30 prenyl side-chain, can accommodate a long prenyl donor substrate because it has a lateral portal through which the long prenyl chain protrudes into the hydrophobic region of a cell membrane (20). Unlike UbiA that accepts a small aromatic prenyl acceptor substrate, however, DGFGP synthase utilizes two large substrates, both of which contain a prenyl group. It is unclear whether both of the substrates are accommodated in the active pocket of the enzyme, or if one or two prenyl groups protrude into the cell membrane. Considering the results from the *in vitro* assay that demonstrated the stricter preference of the enzyme for prenyl donors over prenyl acceptors, the prenyl chain of the prenyl acceptor substrate possibly extends through the lateral portal. Given the fact that HepGP was not accepted even when the donor was GFPP, however, the substrate recognition mechanism is probably more complicated.

Furthermore, we succeeded in constructing an artificial biosynthetic pathway for hyperextended C25,C30-archaeal membrane lipids in *E. coli* cells. Some of the produced lipids had a phosphoglycerol head group. This modification of the polar head group was also observed when other types of archaeal membrane lipids were produced in *E. coli* (15, 28, 32–35). Because such modification occurs at the cell membrane, at least part of the produced C25,C30-archaeal membrane lipids are considered to be included in the cell membrane of *E. coli*. It should be noted that double bonds remain in the produced C25,C30-archaeal membrane lipids. Archaeal lipids possessing unsaturated isoprenoid chains are reportedly quite rare in archaea, except for a hyperthermophilic methanogen, *Methanopyrus kandleri*, and a few examples of psychrophilic archaea (36–38). In a future study, we will attempt to saturate the lipids. To date, *E. coli* strains have been constructed with the ability to produce various archaeal membrane lipids that possess C20,C20-, hydroxylated C20,C20-, or C25,C25-core structures (15, 28, 32–34, 39). Caforio *et al.* (35) recently reported the construction of an *E. coli* strain that produced *sn*-2,3-(digeranylgeranyl)glycerol-based lipids as main components of the cell membrane, constituting up to 20–30% of whole lipids. Intriguingly, the strain had a slightly more robust cell membrane, which demonstrated that the addition of archaeal lipids could affect the properties of the bacterial cell membrane. The present study is the first to construct an *E. coli* strain by synthesizing hyperextended archaeal membrane lipids. Boosting the production of this lipid will elucidate the effect of producing hyperextended lipids on *E. coli* cells. Moreover, by comparing the phenotype of the *E. coli* strain producing C25,C30-archaeal membrane lipids with those of strains producing C20,C20- or C25,C25-lipids, we should gain a better understanding of how the chain-lengths of diether archaeal membrane lipids confer a tolerance of harsh environments and why such lipids have not been discovered in nature.

## Funding

This work was supported by JSPS KAKENHI [18K19170, 19H04651 and 20H02899 to H.H.]; by grants-in-aid from the Institute for fermentation, Osaka, the Noda Institute for Scientific Research, and the Nagase Scientific Technology Foundation to H.H.; and by JSPS KAKENHI [19J21282 to R.Y.].

*Conflict of interest statement*. None declared.

## Supplementary Material

ysaa018_Supplementary_Data
